# Using practice effects for targeted trials or sub-group analysis in Alzheimer’s disease: How practice effects predict change over time

**DOI:** 10.1371/journal.pone.0228064

**Published:** 2020-02-21

**Authors:** Guoqiao Wang, Richard E. Kennedy, Terry E. Goldberg, Mackenzie E. Fowler, Gary R. Cutter, Lon S. Schneider

**Affiliations:** 1 Division of Biostatistics, Washington University in St. Louis, St. Louis, Missouri, United States of America; 2 Comprehensive Center of Aging Health, University of Alabama at Birmingham, Birmingham, Alabama, United States of America; 3 Department of Psychiatry, Columbia University, New York, New York, United States of America; 4 Department of Epidemiology, University of Alabama at Birmingham, Birmingham, Alabama, United States of America; 5 Department of Biostatistics, University of Alabama at Birmingham, Birmingham, Alabama, United States of America; 6 Department of Psychiatry and The Behavioral Sciences, Keck School of Medicine, University of Southern California, Los Angeles, California, United States of America; Appalachian State University, UNITED STATES

## Abstract

**Objective:**

To describe the presence of practice effects in persons with Alzheimer disease (AD) or mild cognitive impairment (MCI) and to evaluate how practice effects affect cognitive progression and the outcome of clinical trials.

**Methods:**

Using data from a meta-database consisting of 18 studies including participants from the Alzheimer disease Cooperative Study (ADCS) and the Alzheimer Disease Neuroimaging Initiative (ADNI) with ADAS-Cog_11_ as the primary outcome, we defined practice effects based on the improvement in the first two ADAS-Cog_11_ scores and then estimated the presence of practice effects and compared the cognitive progression between participants with and without practice effects. The robustness of practice effects was investigated using CDR SB, an outcome independent the definition itself. Furthermore, we evaluated how practice effects can affect sample size estimation.

**Results:**

The overall percent of practice effects for AD participants was 39.0% and 53.3% for MCI participants. For AD studies, the mean change from baseline to 2 years was 12.8 points for the non-practice effects group vs 7.4 for the practice effects group; whereas for MCI studies, it was 4.1 for non-practice effects group vs 0.2 for the practice effects group. AD participants without practice effects progressed 0.9 points faster than those with practice effects over a period of 2 years in CDR-SB; whereas for MCI participants, the difference is 0.7 points. The sample sizes can be different by over 35% when estimated based on participants with/without practice effects.

**Conclusion:**

Practice effects were prevalent and robust in persons with AD or MCI and affected the cognitive progression and sample size estimation. Planning of future AD or MCI clinical trials should account for practice effects to avoid underpower or considers target trials or stratification analysis based on practice effects.

## Introduction

Despite the increase in sample size and duration of Alzheimer’s disease (AD) clinical trials in efforts to detect small clinical effects, recent phase 3 trials failed to detect effective treatments [1–4]. In order to reduce sample sizes and improve efficacy for AD trials, targeted trials or sub-group selection trials which select participants based on genotype (e.g. APOE), cognitive status (e.g. Mini-Mental State Examination (MMSE)), and/or AD biomarkers (e.g. CSF Aβ and tau, or amyloid PET), have been recommended [5–7]. But targeted trials or sub-group selection based on genotype APOE ε4 or cognitive status were shown to possibly be inefficient through simulation using pooled AD trial data [8, 9]. Similarly, two recent phase 3 targeted trials of bapineuzumab selected 1331 AD participants who were not APOE ε4 carriers and 1121 who were APOE ε4 carriers based on the hypothesis that non-carriers were responsive to bapineuzumab, whereas carriers were not, did not achieve the expected reduction in AD assessment scale-cognitive subscale (ADAS-Cog_11_). Thus either the treatment is ineffective or this is consistent with the conclusion that designing targeted trial or sub-group selection based on genotype APOE ε4 may not work unless there was a sufficiently large differential treatment effect between the APOE ε4 groups [8, 9]. These targeted trials or sub-group selection rely on assumptions that certain genotypes and biomarkers predict more rapid change in ADAS-Cog_11_ or other clinical outcomes, making it easier to see differences in treatment effects. These assumptions are made, however, despite the uncertainty of how well established the relationship is, and the fact that the biomarkers have often diverged from hypothesized cognitive results [10].

Adding to these shortcomings is the imperfect measurement of cognitive functions. One prevalent issue is most cognitive tests have learning curves or practice effects [11]. Thus, it may be worthwhile to consider the presence or absence of practice effects as a classifier in clinical trials. Practice effects are defined as the improvement in serial cognitive tests with the same or similar test materials [12]. Practice effects can be identified in subjects with mild cognitive impairment (MCI) or AD, and improvement in serial cognitive tests due to practice effects can lead to an effect size (ratio of change in test-retest scores to its standard error) in low to medium effect size range [13].

Several findings have emerged to support using practice effects as a classifier in clinical trials. For example, short-term practice effects are correlated to AD biomarkers such as amyloid deposition and brain hypometabolism [12, 14]. Practice effects may distinguish cognitively intact elders from those with MCI in that the former demonstrate larger practice effects whereas the latter may or may not [15]. In a study focusing on amnestic MCI, practice effects were shown to predict cognitive outcome after one year [16]. However, these findings were based on small samples and thus needed validation in larger cohorts [12, 14–16]. Furthermore, it is unknown how practice effects affect the change in serial cognitive tests (such as the rate of change and its variability) in longitudinal studies, and how it could be utilized in AD clinical trials. We aim to investigate the applicability of using practice effects as a classifier for designing targeted MCI or AD clinical trials or sub-group analyses using a meta-database of 18 studies.

## Method

### Data sources

Subjects were from a meta-database consisting of 18 studies including participants from the Alzheimer’s Disease Cooperative Study (ADCS) and the Alzheimer’s Disease Neuroimaging Initiative (ADNI), representing both clinical trials and observational studies in AD, MCI, and normal individuals [17]. Of these 18 studies, 10 which had ADAS-cog scores with follow-up duration over 12 months could be used for this study (9 AD studies and 1 MCI study). After exclusion of those with missing values at baseline/screening, or at the first visit after baseline/screening, or at both, a total of 2499 AD participants and 1191 MCI participants were included in this study. In doing so, practice effects were defined for the same time interval within each study. All data obtained from the Alzheimer’s Disease Cooperative Study were deidentified and anonymized before access.

### Outcomes

The 11-item ADAS-Cog was the primary outcome measure for most of these trials and used in all of the trials. It is a brief cognitive battery that evaluates memory, reasoning, orientation, praxis, language, and word finding difficulty and is scored as a composite from 0 to 70 errors with higher scores indicating worse impairment [18]. The clinical dementia rating-sum of boxes (CDR-SB) was the secondary outcome, which evaluates memory, orientation, judgment and problem solving, community affairs, home and hobbies, and personal care. Each item or domain is scored 0, 0.5, 1, 2, or 3 in increasing severity (the personal care item does not allow a 0.5 score). It too is scored as a composite rating, the six domains summed to a score from 0 to 18 with higher score indicating worse impairment [19].

### Definition of practice effects

Hassenstab et al. defined practice effect using the slope of the first three yearly measurements [20]. As they and others observed, practice effects occur mostly between the first and second administration of the test [20–23]. Thus, we defined practice effects based on the first two measurements. This method was chosen also due to its easy implementation in a real clinical trial setting, where simple and non-model-based methods are preferred to avoid any selection bias. A participant was considered demonstrating practice effects if this individual’s first ADAS-Cog_11_ score (at screening or at baseline) is higher than or equal to the second, meaning improvement or lack of worsening in cognitive scores is observed. To avoid defining false practice effects due to random measurement error, we conducted sensitivity analysis where the practice effects was defined as a minimum of 2 points improvement in ADAS-Cog_11_ from the first to the second assessment.

### Robustness of practice effects

The practice effects were defined using ADAS-Cog_11_, to evaluate the robustness of practice effects, we chose a secondary outcome CDR-SB. CDR-SB offered multiple advantages to test the robustness. First, it was independent of the definition of practice effects which was based on ADAS-Cog_11_ although it is not completely immune to practice effects. Second, very few items in CDR-SB are overtly susceptible to practice effects and the ratings themselves are subjective, making any differences observed in CDR-SB between the practice effects groups most likely to be true differences rather than due to regression to the mean or random errors. Third, almost every study with ADAS-Cog_11_ also had CDR-SB, minimizing the impact of sample size difference between these two outcomes.

### Statistical analysis

We characterized the study cohort using descriptive statistics and calculated the prevalence of practice effects for each trial. Baseline ADAS-Cog_11_, age, and the years of education were compared using ANOVA. The association between practice effects and age, sex, education, and baseline ADAS-Cog_11_ was investigated using multiple variable logistic regressions for pooled AD participants and pooled MCI participants, respectively.

The mixed model for repeated measure (MMRM) with time as a categorical variable was used for the primary analyses in three different ways. (i) It was applied to each clinical trial, separately, to estimate the mean over time and compare the change from baseline in ADAS-Cog_11_ between the practice effects group and the non-practice effects group. (ii) Then the individual ADAS-Cog_11_ scores of the same visits in the 10 AD studies were pooled together for another MMRM analysis for AD participants and MCI participants, respectively. Specifically, the scores at baseline, 6 months, 12 months, 18 months, and 24 months were pooled. (iii) Finally, MMRM was applied in the same way as in (ii) with CDR-SB as the dependent variable. The MMRM model was constructed with group effect (practice effects group vs non-practice effects group), time effect, and group by time interaction as the fixed effects with an unstructured covariance matrix.

Sample size estimation were conducted for subjects with and without practice effects based on pooled AD studies and MCI studies, respectively. Similar to previous trials [1, 3], we first estimated the mean (SD) of the 2-year change from baseline, then under the assumption of equal variance among the treatment groups, we estimate the sample size for a given treatment effect using two-sample t-tests and equal randomization ratio. The treatment effect is either an absolute reduction or a percentage reduction in the mean change from baseline.

Analyses were conducted using SAS 9.4 (SAS Institute Inc., Cary, NC).

## Results

### Practice effects and ADAS-Cog_11_ progression over time

Participants with practice effects were observed in all 10 studies (ADNI AD and ADNI MCI were considered as 1 study) ranging from 26.9% to 57.7% (Table 1). The overall percent of practice effects for AD participants was 39.0% and 53.3% for MCI participants. The percent of practice effects is 42.1% in pooled treatment arm and is 44.2% in the pooled placebo arm (p = 0.19). Participants with and without practice effects had similar number of years of education in all 10 studies, and significantly differed in age in 3 studies (Table 1). Significantly higher mean baseline ADAS-Cog_11_ levels were found for participants with practice effects in 6 studies (again ADNI AD and ADNI MCI were considered as 1 study) (Table 1). For AD participants, practice effects were only associated with baseline ADAS-Cog_11_; for a 1 point increase in baseline ADAS-cog, the odds of demonstrating practice effect increased by 1.6% (odds ratio: 1.016 [95% CI: 1.008–1.023]). For MCI participants, practice effects were associated with baseline ADAS-Cog_11_ and age. For a 1 point increase in baseline ADAS-Cog_11_, the odds of demonstrating practice effects increased by 10.2% (odds ratio: 1.102 [95% CI: 1.070–1.135]); whereas for 1 year increase in age, the odds of demonstrating practice effect decreased by 4% (odds ratio: 0.960 [95% CI: 0.944–0.977]).

**Table 1:** Comparison between practice effects group and non-practice effects group.

Study	Total Duration (months)	Duration Between baseline and 2^nd^ measurement (months)	N (%) with Practice Effects				Baseline ADAS-cog_11_			Baseline Age, y			Education, y		
			Treatment	Placebo	p	N/ Total, (%)	PE**	Non-PE	p	PE**	Non-PE	p	PE**	Non-PE	p
ADNI AD	24	6	NA	60/176 (34.1)	NA	60/176 (34.1)	20.0	17.9	0.0439	76.5	74.5	0.0991	14.7	14.8	0.92
DHA	18	6	76/219 (34.7)	45/151 (29.8)	0.32	121/370 (32.7)	23.5	23.7	0.8051	75.5	76.0	0.6102	14.5	14.2	0.3810
ES	15	2	34/78 (43.6)	13/38 (34.2)	0.33	47/116 (40.5)	24.2	23.6	0.7029	75.0	75.4	0.7118	12.4	12.2	0.7419
HC	18	6	99/230 (43.0)	59/160 (36.9)	0.22	158/390 (40.5)	23.8	21.4	0.0074	76.5	76.3	0.7426	14.0	13.8	0.5173
LL	18	6	74/193 (38.3)	78/188 (41.5)	0.53	152/381 (39.9)	25.5	23.1	0.0235	74.6	73.8	0.4183	14.0	14.4	0.2177
NS	14	3	83/209 (39.7)	43/103 (41.8)	0.73	126/312 (40.4)	22.9	23.9	0.3715	74.5	73.5	0.2501	14.2	13.9	0.3756
PR	16	28 weeks	32/69 (46.4)	31/69 (44.9)	0.86	63/138 (45.7)	24.1	19.9	0.0121	70.6	73.8	0.0134	14.2	13.9	0.6091
SL	24	1	103/236 (43.6)	31/76 (40.8)	0.66	134/312 (43.0)	38.3	38.9	0.5907	72.8	72.2	0.5360	12.6	12.4	0.7548
VN	26	6	26/113 (23.0)	35/114 (30.7)	0.19	61/227 (26.9)	31.2	27.4	0.0036	75.4	75.7	0.8011	13.3	13.9	0.2012
ADNI MCI	24	6	NA	175/386 (45.3)	NA	175/386 (45.3)	12.5	10.8	0.0003	73.9	75.6	0.0241	15.5	15.8	0.45
MCI	36	3	258/457 (56.5)	145/241 (60.2)	0.35	403/698 (57.7)	11.9	10.2	<.0001	72.0	73.2	0.0240	14.7	14.7	0.996

Baseline ADAS-cog scores for participants with practice effects were significantly higher than for those without in 6 trials but were similar in the other 4 trials. Age and the years of education were mostly similar.

PE: practice effects, Non-PE: Non-practice effects

The estimated mean ADAS-Cog_11_ score for patients with practice effects was generally higher at baseline than for those without practice effects at baseline, but lower in post baseline visits in all but one study regardless of the similarity in the mean baseline ADAS-Cog_11_ (S1 and S2 Figs). Generally, the mean post-baseline trajectories over time demonstrated a parallel pattern (S1 and S2 Figs). When estimating the change from baseline, the non-practice effects group in the AD studies worsened 5.3 to 10.7 points by 1.5 years and 8.9 to 17.5 points by 2 years; whereas the practice effects group worsened 3.0 to 10.2 points by 1.5 years and 5.5 to 12.5 points by 2 years (S3 and S4 Figs). In the MCI studies, the non-practice effects group worsened 3.1 to 4.1 points by 1.5 years and 5.8 to 7.1 points by 3 years; whereas the practice effects group improved by 0.1 to 0.3 points by 1.5 years and worsened 2.4 to 3.0 points by 3 years.

**Fig 1:**
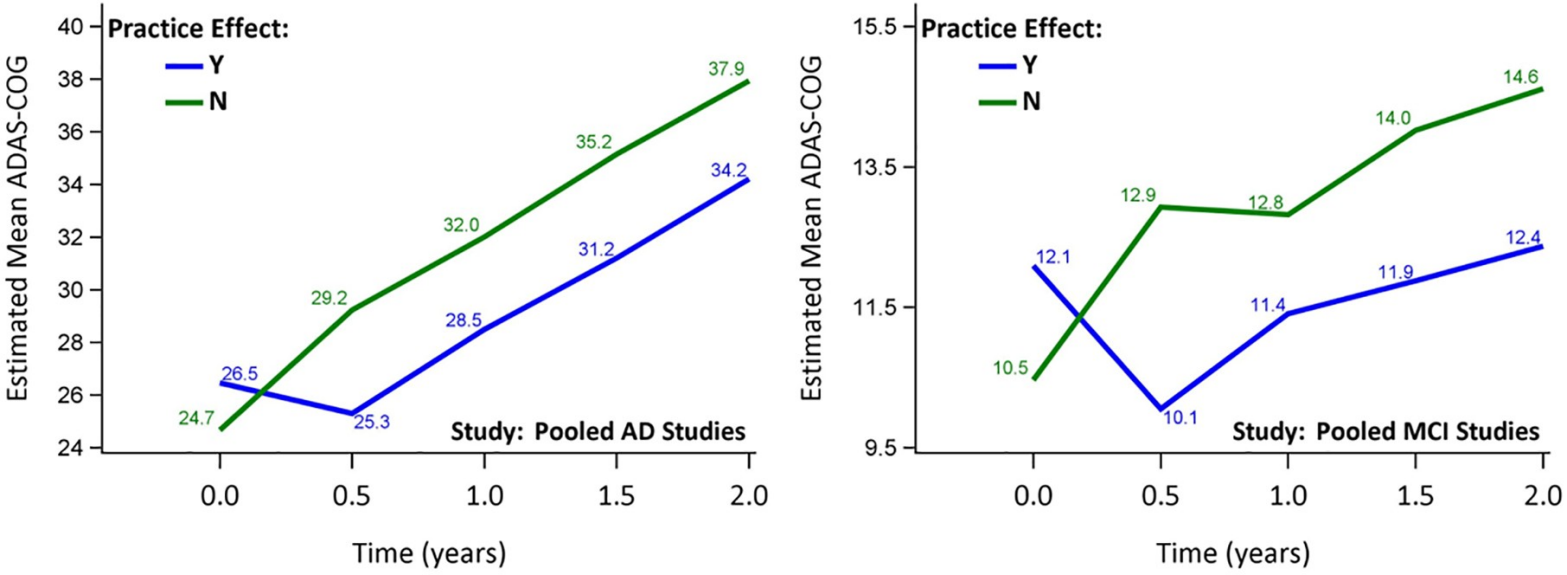
Estimated mean ADAS-Cog_11_ over time for pooled AD/MCI studies. Similar results to those individual studies were observed for the mean ADAS-Cog_11_ over time.

After pooling the data, similar results were observed for the mean ADAS-Cog_11_ over time (Fig 1). For pooled AD studies, the mean change from baseline to 2 years was 12.8 points for the non-practice effects group vs 7.4 for the practice effects group; whereas for MCI studies, it was 4.1 for non-practice effects group vs 0.2 for the practice effects group (S5 Fig).

### Practice effects affect change in CDR-SB

**Fig 2:**
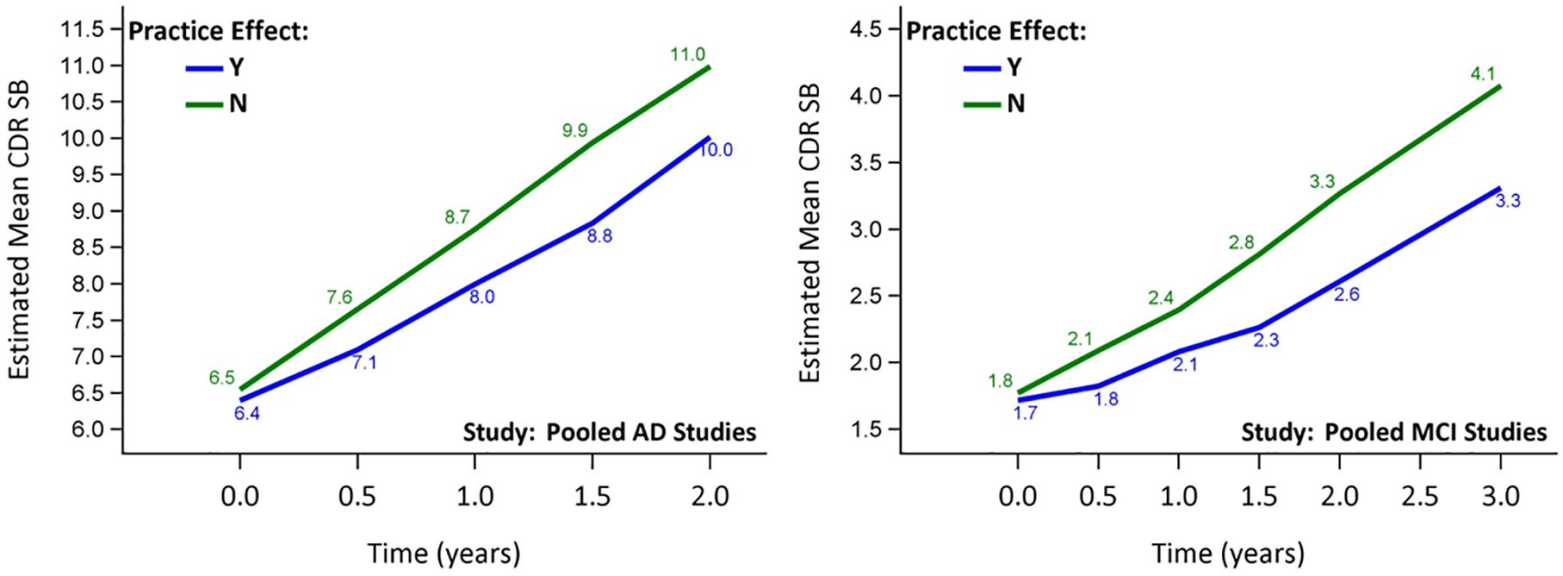
CDR-SB progression over time for pooled AD studies and pooled MCI studies. For both AD and MCI studies, the non-practice effects group progressed faster.

With the same practice effects groups defined by ADAS-Cog_11_ scores, we investigated the progression using an alternative outcome, the CDR-SB, pooling data for AD and for MCI, respectively. This would avoid potential problems with defining practice effects based on the same instrument used for the trial outcome. The practice effects group had slightly lower mean CDR-SB than the non-practice effects group at baseline, but the discrepancy increased from 0.1 at baseline to 1.0 over a period of 2 years with faster progression in the latter (p = 0.0015 for the interaction between practice effects group and time) (Fig 2). Similar but smaller discrepancy was also observed for pooled MCI studies, and the discrepancy increased from 0.1 to 0.8 over a period of 36 months with faster progression observed in the non-practice effects group (p = 0.0001 for the interaction between practice effects group and time) (Fig 2).

### Practice effects affect sample sizes

**Fig 3:**
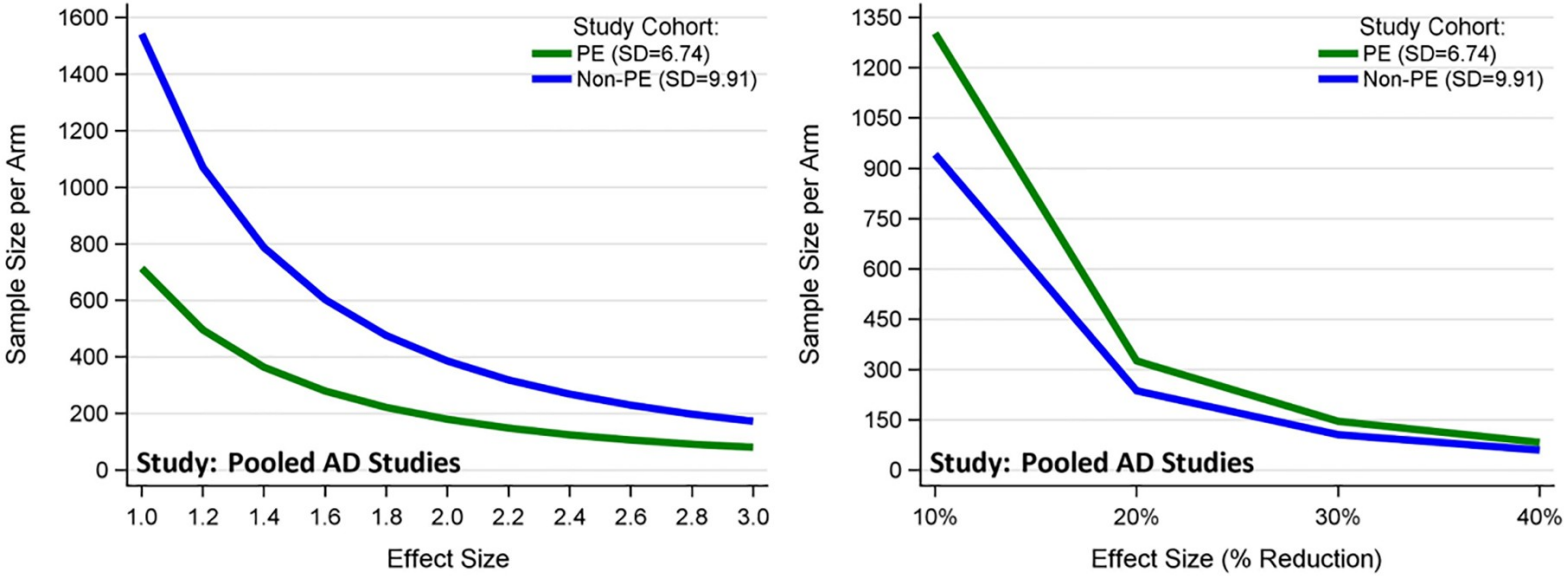
Sample size comparison for different sample size estimation methods (based on 80% power, 5% type I error, two-sided test, equal duration). The left panel estimated the sample size by assuming the same minimum clinical meaningful difference; whereas the right panel by assuming the same percentage reduction in the progression of the placebo group. Sample size were estimated based on two-sample t-tests.

When planning AD clinical trials, the sample size can be estimated in two ways. One is to estimate the mean (SD) of the change from baseline to the end of study for the primary endpoint using pilot studies or previously published studies; then a minimum clinical meaningful difference (or mean clinically important difference) is chosen to calculate the sample size. For example, in the EXPEDITION and EXPEDITION2 trials, the minimum clinical meaningful difference was chosen to be 1.8 points at 18 months on the ADAS-Cog_11_ [1] in patients with mild to moderate AD; whereas subsequently in the EXPEDITION3 trial, the minimum clinical meaningful difference was chosen to be 1.5 points at 18 months using the expanded ADAS-Cog_14_ [3], and in a mild AD subset. Because the practice effects group has smaller variance than the non-practice effects group, it has smaller sample size when the same minimum clinical meaningful difference is used for both groups (Fig 3, left panel). With all things being equal (e.g. 80% power, 5% type I error, two-sided test, equal duration), the sample size for the practice effects cohort is 46% of that for the non-practice effects cohort. Another common way to consider sample size is to assume that the treatment effect leads to a certain percentage reduction for the treatment group compared with the progression of the placebo group. Based on the pooled AD studies, the mean (SD) decline in 2 years is 7.4 (6.74) for the practice effects group and 12.8 (9.91) for the non-practice effects group. A 10% reduction will lead to an absolute reduction of 0.74 and 1.28 for the practice and non-practice effects groups, respectively. The sample sizes estimated from this perspective are shown in the right panel of Fig 3. When all things being equal, the sample size for the practice effects cohort is 138% of that for the non-practice effects cohort.

### Sensitivity analysis using new practice effects group

Using the redefined practice effects groups (improvement of at least 2 points in ADAS-Cog_11_), we re-ran all the analysis and obtained similar results in terms of the progression in CDR-SB, the change over time in ADAS-Cog_11_, and the variance of each group. Because some practice effects subjects under the first definition now belonged to the non-practice effects group under the second definition, the difference between the two groups for the second definition became smaller (S6 and S7 Figs).

## Discussion

Using a meta-database with over 3000 participants, we demonstrated that those with practice effects showed substantially less worsening on the ADAS-Cog_11_ over the course of 3 to 24 months than those without. The difference in the rate of change in ADAS-Cog_11_ between those with practice effects and those without is even larger than the difference expected between experimental drugs and placebo in current trials or from the observed effects of marketed cholinesterase inhibitors compared with placebo. The 3- to 5-point difference across 18 months of trial duration is 2 to 3 times the differences planned in the current clinical trials of experimental drugs for mild or moderate dementia due to AD for early AD trials [1, 3, 4, 24], or the actual difference in marketed cholinesterase inhibitors trials compared with placebo [25, 26].

Varying the mixture of participants with practice effects and those without in AD trials, therefore, may have significant impact on the outcome of such trials due to the large differences in sample sizes. For example, assuming that the percentage reduction is the same, the change in ADAS-Cog_11_ in the practice effects group is 2 points and in the non-practice effects group is 4 points with a common SD 8, then a 40% reduction would lead to an effect size of 0.1 (= 2*40%/8) for the practice effects group and an effect size of 0.2 (= 4*40%/8) for the non-practice effects group. Given the same sample size, such differences in the change from baseline and in the resulting effect sizes may be crucial in determining the success of AD trials. On the other hand, if the same minimum clinical meaningful difference is used for each group (meaning different percentage reductions are assumed for each group), then the practice effects group requires a much smaller sample size than the non-practice effects group. However, it is unclear which group of participants is more likely to respond to an experimental drug. For example, if the non-practice effects participants are associated with more rapidly progressive disease that is treatment-resistant, while the practice effects participants are more slowly progressive but treatment-responsive, then a larger trial with practice effects participants would be required to detect treatment effects. Moreover, absolute cutoff scores in ADAS-Cog_11_ change have been recommended to interpret treatment effects. For example, 1.5- to 4-point differences in ADAS-Cog_11_ (11 items or 14 items) over 6 to 18 months have been considered by some to be minimally clinically relevant [1, 3, 27–29]. Differences in the change of ADAS-Cog_11_ from baseline between the practice effects and non-practice effects participants suggest that these cutoff scores may miss a number of practice effects participants showing a significant change that is less than these thresholds.

Instead of relying on presumed relationships between genotypes or biomarkers and the ADAS-Cog_11_ which itself will vary by illness stage, using practice effects provides a direct way for designing targeted trials or sub-group analyses based on the ADAS-Cog_11_ sample distribution for clinical trials. It also offers an alternative standard to measure disease severity based on the trial participants’ learning ability. Participants who learn or improve their ADAS-Cog_11_ scores are more likely to be less cognitively impaired than the non-practice effects participants, even within a narrowly defined study sample. The difference in practice effects can subsequently be taken into account in the statistical analysis or used for stratification of subjects to enhance an efficacy signal [10]. Furthermore, instead of trying to eliminate or alleviate the impact of practice effects through various strategies [13], using practice effects to design targeted trials or sub-group analyses provides an alternative to take advantage of practice effects for better signal detection. We cannot, however, make a conclusive recommendation regarding who should be included in targeted trials (non-practice effects subjects vs practice effects subjects) since no data or published research so far have shown which group is more responsive to the active treatments although a study has shown subjects with practice effects benefitted more from a cognitive intervention [30].

There is a prevalent opinion in AD research community that participants should be treated in clinical trials during the earlier stages of the illness in order to maximize the treatment benefits. Consequently, sub-group analyses based on the stages of illness have been conducted (e.g. mild AD vs moderate AD) [1]. The MMSE at visit 1 was commonly used to differentiate mild AD (e.g. MMSE score of 20 to 26 or 21 to 26) from moderate AD (e.g. MMSE score of 16 to 19), though the thresholds may vary [1, 2, 31]. Given the correlation between ADAS-Cog_11_ and MMSE and the similarity in their primary cognitive functions [32], the observed practice effects in ADAS-Cog_11_ implicate the potential inaccuracy of categorizing disease stages by a single measurements. In future studies, it may be helpful to use the average of the first two measurements for categorizing disease stages.

In our analyses, we observed that the strongest predictor of the practice effects group was the severity of cognitive impairment on the ADAS-Cog_11_. This tells us that the information that is classifying individuals as having practice effects may be due to regression toward the mean [33]. Although this does not imply that those unable to mount practice effects are not declining faster or reaching critical values sooner. To attempt to assess if the non-practice effects are truly farther down the cognitive decline scale versus appearing in the group because of regression toward the mean, we examined a more broadly based, summary clinical outcome, the CDR-SB, that in addition to assessing memory, orientation, judgment and problem-solving, requires an assessment of a patient’s participation in community affairs, home and hobbies, and personal care. Although regression toward the mean could still occur, we expect this effect would be attenuated on a clinical outcome rating, we could examine if the group with a more rapid decline posited as those who truly cannot learn could be seen once this attenuated regression toward the mean were used. In these analyses, we found a group by time interaction. This suggests that the practice effects are present within the cohort as we defined them and are not completely due to regression toward the mean. To evaluate the effects of misclassification of practice effects due to measurement errors in ADAS-Cog_11_, we re-classified practice effects groups using a minimum clinical meaningful threshold of 2 points. Results based on this classification are very similar and reinforce the robustness of practice effects.

Potential limitations to this study include that the practice effects were defined based on two assessments collected over a variety of time intervals ranging from 28 weeks to 6 months. Although it would be ideal if all the intervals were the same, the consistent findings from each individual study and from the pooled studies alleviated the effects of unequal intervals. In a planned clinical trial, these two assessments can be collected at the screening and at the baseline; or consecutively at the baseline with a few hours/days apart before the administration of experimental drugs. How the results from this study may apply to these scenarios warrant further validation. Second, our findings are based on ADAS-Cog_11_ and need to be tested with different cognitive outcomes. Additionally, using ADAS-Cog_11_ may underestimate the practice effect due to its lack of sensitivity to detect changes in preclinical AD stage. The use of measurements with greater sensitivity to change would be expected to lead to even greater power to detect changes in cognition and function over time. Third, from a pragmatic perspective of conducting a clinical trial, we employed the most straightforward method to define the practice effects by using the first two assessments. Although other methods such as the complex standardized regression-based method can be used [34], these methods involve the use of demographic variables and may cause selection bias. Finally, the practice effects can be altered by different cognitive domains, demographic variables, subject variables, retest interval, etc., our analyses did not address these issues.

Although, these findings might be confirmed in other pooled databases of cohort studies or clinical trials, it appears that practice effects can independently reflect participants’ true cognitive status and lead to significant difference in cognitive decline between participants with and without practice effects. Trial planning without considering practice effects may result in inefficient trials with insufficient power. It may be worthwhile to utilize practice effects in clinical trials by beginning with planning sub-group analysis in future studies or even targeted trials.

## Supporting information

S1 Fig Estimated mean ADAS-Cog_11_ over time for AD studies. The practice effects group progressed more slowly than the non-practice effects group in all but one study.
(DOCX)

S2 Fig Estimated mean ADAS-Cog_11_ over time for MCI studies. The practice effects group progressed more slowly than the non-practice effects group.
(DOCX)

S3 Fig Estimated mean ADAS-Cog_11_ change over time for AD studies. The practice effects group progressed more slowly than the non-practice effects group in all studies.
(DOCX)

S4 Fig Estimated mean ADAS-Cog_11_ change over time for MCI studies. The practice effects group progressed more slowly than the non-practice effects group.
(DOCX)

S5 Fig Estimated mean ADAS-Cog_11_ change over time for pooled AD/MCI studies. Similar results to those individual studies were observed for the mean ADAS-Cog_11_ change over time.
(DOCX)

S6 Fig CDR-SB progression over time for pooled AD studies and pooled MCI studies by new practice effects groups. For both AD and MCI studies, the non-practice effects group declined faster.
(DOCX)

S7 Fig Estimated mean ADAS-Cog_11_ change over time for pooled AD/MCI studies by new practice effects groups. (DOCX)

## References

[1] Doody RS, Thomas RG, Farlow M, Iwatsubo T, Vellas B, Joffe S, et al Phase 3 trials of solanezumab for mild-to-moderate Alzheimer’s disease. New England Journal of Medicine. 2014;370:311–21. 10.1056/NEJMoa1312889 24450890

[2] Salloway S, Sperling R, Fox NC, Blennow K, Klunk W, Raskind M, et al Two phase 3 trials of bapineuzumab in mild-to-moderate Alzheimer’s disease. New England Journal of Medicine. 2014;370:322–33. 10.1056/NEJMoa1304839 24450891 PMC4159618

[3] Honig LS, Vellas B, Woodward M, Boada M, Bullock R, Borrie M, et al Trial of solanezumab for mild dementia due to Alzheimer’s disease. New England Journal of Medicine. 2018;378:321–30. 10.1056/NEJMoa1705971 29365294

[4] Egan MF, Kost J, Tariot PN, Aisen PS, Cummings JL, Vellas B, et al Randomized trial of verubecestat for mild-to-moderate Alzheimer’s disease. New England Journal of Medicine. 2018;378:1691–703. 10.1056/NEJMoa1706441 29719179 PMC6776074

[5] Sperling RA, Rentz DM, Johnson KA, Karlawish J, Donohue M, Salmon DP, et al The A4 study: stopping AD before symptoms begin? Science translational medicine. 2014;6:228fs13–fs13. 10.1126/scitranslmed.3007941 24648338 PMC4049292

[6] Cummings JL. Alzheimer’s disease clinical trials: changing the paradigm. Current psychiatry reports. 2011;13:437–42. 10.1007/s11920-011-0234-y 22052382

[7] Ito K, Corrigan B, Zhao Q, French J, Miller R, Soares H, et al Disease progression model for cognitive deterioration from Alzheimer’s Disease Neuroimaging Initiative database. Alzheimer’s & Dementia. 2011;7:151–60. 10.1016/j.jalz.2010.03.018 20810324

[8] Kennedy RE, Cutter GR, Schneider LS. Effect of APOE genotype status on targeted clinical trials outcomes and efficiency in dementia and mild cognitive impairment resulting from Alzheimer’s disease. Alzheimer’s & Dementia. 2014;10:349–59. 10.1016/j.jalz.2013.03.003 PMC3900604 23712001

[9] Kennedy RE, Cutter GR, Wang G, Schneider LS. Using baseline cognitive severity for enriching Alzheimer’s disease clinical trials: How does Mini-Mental State Examination predict rate of change? Alzheimer’s & Dementia: Translational Research & Clinical Interventions. 10.1016/j.trci.2015.03.001 PMC5040516 27695707

[10] Vellas B, Carrillo MC, Sampaio C, Brashear HR, Siemers E, Hampel H, et al Designing drug trials for Alzheimer’s disease: what we have learned from the release of the phase III antibody trials: a report from the EU/US/CTAD Task Force. Alzheimer’s & Dementia. 2013;9:438–44. 10.1016/j.jalz.2013.03.007 23809364

[11] Goldberg TE, Goldman RS, Burdick KE, Malhotra AK, Lencz T, Patel RC, et al Cognitive improvement after treatment with second-generation antipsychotic medications in first-episode schizophrenia: is it a practice effect? Archives of general psychiatry. 2007;64:1115–22. 10.1001/archpsyc.64.10.1115 17909123

[12] Duff K, Foster NL, Hoffman JM. Practice effects and amyloid deposition: Preliminary data on a method for enriching samples in clinical trials. Alzheimer Disease & Associated Disorders. 2014;28:247–52. 24614265 10.1097/WAD.0000000000000021 PMC4139470

[13] Goldberg TE, Harvey PD, Wesnes KA, Snyder PJ, Schneider LS. Practice effects due to serial cognitive assessment: Implications for preclinical Alzheimer’s disease randomized controlled trials. Alzheimer’s & Dementia: Diagnosis, Assessment & Disease Monitoring. 2015;1:103–11. 10.1016/j.dadm.2014.11.003 PMC4876902 27239497

[14] Duff K, Horn KP, Foster NL, Hoffman JM. Short-Term Practice Effects and Brain Hypometabolism: Preliminary Data from an FDG PET Study. Archives of Clinical Neuropsychology. 2015;30:264–70. 10.1093/arclin/acv018 25908614 PMC4481562

[15] Duff K, Chelune G, Dennett K. Within-session practice effects in patients referred for suspected dementia. Dementia and geriatric cognitive disorders. 2012;33:245–9. 10.1159/000339268 22813981 PMC3448265

[16] Duff K, Lyketsos CG, Beglinger LJ, Chelune G, Moser DJ, Arndt S, et al Practice effects predict cognitive outcome in amnestic mild cognitive impairment. The American Journal of Geriatric Psychiatry. 2011;19:932–9. 10.1097/JGP.0b013e318209dd3a 22024617 PMC3202689

[17] Schneider LS, Kennedy RE, Cutter GR, Initiative AsDN. Requiring an amyloid-β 1–42 biomarker for prodromal Alzheimer’s disease or mild cognitive impairment does not lead to more efficient clinical trials. Alzheimer’s & Dementia. 2010;6:367–77. 10.1016/j.jalz.2010.07.004 PMC2947209 20813339

[18] Mohs RC, Knopman D, Petersen RC, Ferris SH, Ernesto C, Grundman M, et al Development of cognitive instruments for use in clinical trials of antidementia drugs: additions to the Alzheimer’s Disease Assessment Scale that broaden its scope. Alzheimer Disease & Associated Disorders. 1997;11:13–21. 9236948

[19] Morris JC. The Clinical Dementia Rating (CDR): current version and scoring rules. Neurology. 1993. 10.1212/wnl.43.11.2412-a 8232972

[20] Hassenstab J, Ruvolo D, Jasielec M, Xiong C, Grant E, Morris JC. Absence of Practice Effects in Preclinical Alzheimer’s Disease. 2015. 10.1037/neu0000208 PMC4640964 26011114

[21] CALAMIA M, MARKON K, TRANEL D. Scoring Higher the Second Time Around: Meta-Analyses of Practice Effects in Neuropsychological Assessment. Neuropsychology, development, and cognition Section D, The clinical neuropsychologist. 2012;26:543–70. 10.1080/13854046.2012.680913 22540222

[22] Falleti MG, Maruff P, Collie A, Darby DG. Practice effects associated with the repeated assessment of cognitive function using the CogState battery at 10-minute, one week and one month test-retest intervals. Journal of Clinical and Experimental Neuropsychology. 2006;28:1095–112. 10.1080/13803390500205718 16840238

[23] Collie A, Maruff P, Darby DG, McSTEPHEN M. The effects of practice on the cognitive test performance of neurologically normal individuals assessed at brief test–retest intervals. Journal of the International Neuropsychological Society. 2003;9:419–28. 10.1017/S1355617703930074 12666766

[24] Schneider L, Mangialasche F, Andreasen N, Feldman H, Giacobini E, Jones R, et al Clinical trials and late‐stage drug development for Alzheimer’s disease: an appraisal from 1984 to 2014. Journal of internal medicine. 2014;275:251–83. 10.1111/joim.12191 24605808 PMC3956752

[25] Birks J. Cholinesterase inhibitors for Alzheimer’s disease. The Cochrane Library 2006. 10.1002/14651858.CD005593 PMC9006343 16437532

[26] Takeda A, Loveman E, Clegg A, Kirby J, Picot J, Payne E, et al A systematic review of the clinical effectiveness of donepezil, rivastigmine and galantamine on cognition, quality of life and adverse events in Alzheimer’s disease. International journal of geriatric psychiatry. 2006;21:17–28. 10.1002/gps.1402 16323253

[27] Vellas B, Andrieu S, Sampaio C, Coley N, Wilcock G. Endpoints for trials in Alzheimer’s disease: a European task force consensus. The Lancet Neurology. 2008;7:436–50. 10.1016/S1474-4422(08)70087-5 18420157

[28] Schott J, Schrag A. 024 What is the clinically relevant change on the ADAS-cog? Journal of Neurology, Neurosurgery & Psychiatry. 2012;83:e1–e. 10.1136/jnnp-2011-300881 22019547

[29] Rockwood K, Fay S, Gorman M. The ADAS-cog and clinically meaningful change in the VISTA clinical trial of galantamine for Alzheimer’s disease. International journal of geriatric psychiatry. 2010;25:191–201. 10.1002/gps.2319 19548273

[30] Duff K, Beglinger LJ, Moser DJ, Schultz SK, Paulsen JS. Practice effects and outcome of cognitive training: Preliminary evidence from a memory training course. The American Journal of Geriatric Psychiatry. 2010;18:91 10.1097/jgp.0b013e3181b7ef58 20104658 PMC3640991

[31] Boada M, López O, Núñez L, Szczepiorkowski ZM, Torres M, Grifols C, et al Plasma exchange for Alzheimer’s disease Management by Albumin Replacement (AMBAR) trial: Study design and progress. Alzheimer’s & Dementia: Translational Research & Clinical Interventions. 2019;5:61–9. 10.1016/j.trci.2019.01.001 PMC6395854 30859122

[32] Doraiswamy P, Bieber F, Kaiser L, Krishnan K, Reuning-Scherer J, Gulanski B. The Alzheimer’s disease assessment scale Patterns and predictors of baseline cognitive performance in multicenter Alzheimer’s disease trials. Neurology. 1997;48:1511–7. 10.1212/wnl.48.6.1511 9191757

[33] Nesselroade JR, Stigler SM, Baltes PB. Regression toward the mean and the study of change. Psychological Bulletin. 1980;88:622.

[34] Duff K, Atkinson TJ, Suhrie KR, Dalley BCA, Schaefer SY, Hammers DB. Short-term practice effects in mild cognitive impairment: Evaluating different methods of change. Journal of clinical and experimental neuropsychology. 2017;39:396–407. 10.1080/13803395.2016.1230596 27646966 PMC5738658

