# Supplementary figures and images for: Using practice effects for targeted trials or sub-group analysis in Alzheimer’s disease: How practice effects predict change over time
Source: PLoS One. 2020 Feb 21;15(2):e0228064. doi: 10.1371/journal.pone.0228064 (PMC7034859; doi:10.1371/journal.pone.0228064)

S1 Fig


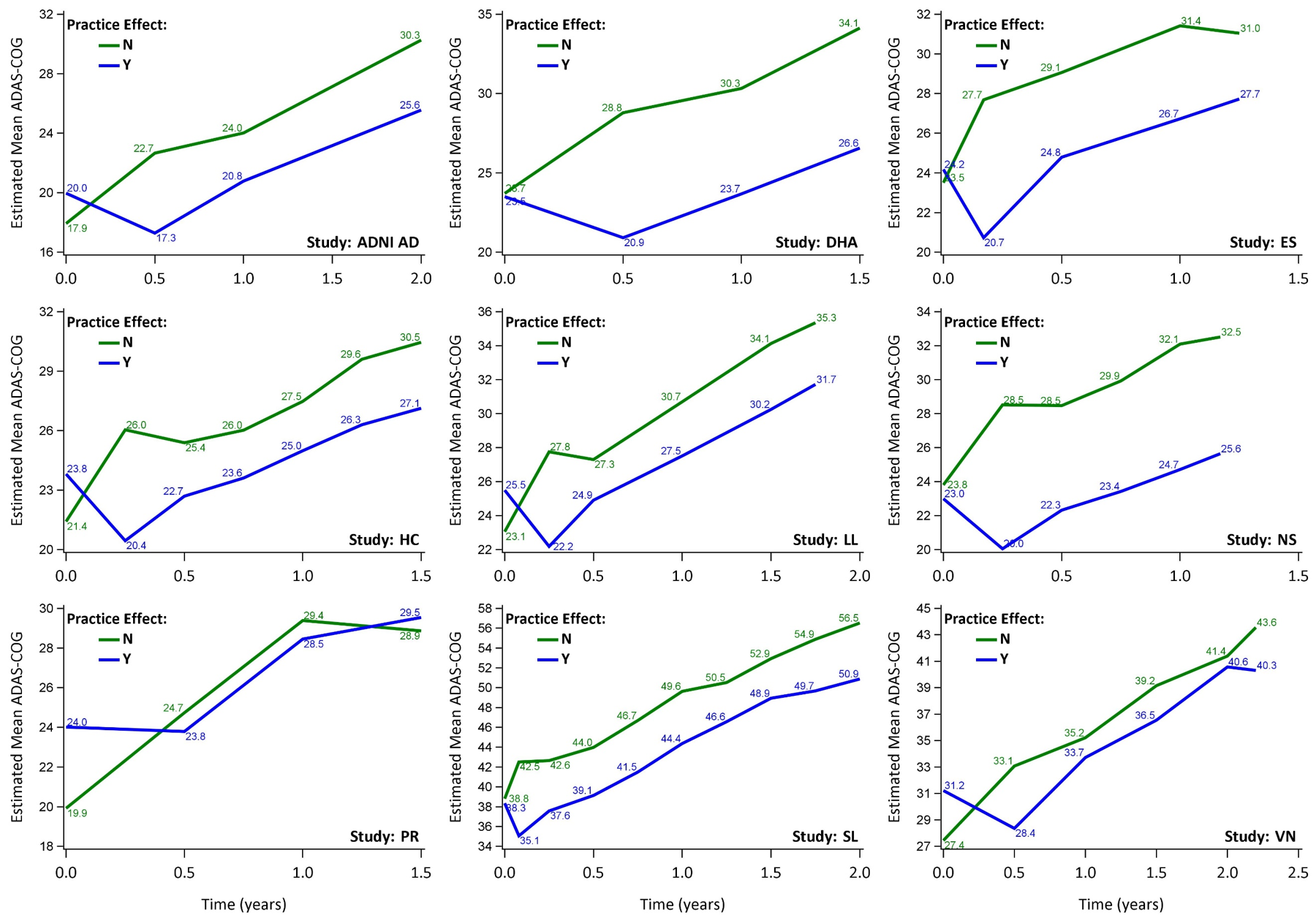

Supplement: S1 Fig — The practice effects group progressed more slowly than the non-practice effects group in all but one study. (DOCX) [file pone.0228064.s001.docx]

S2 Fig


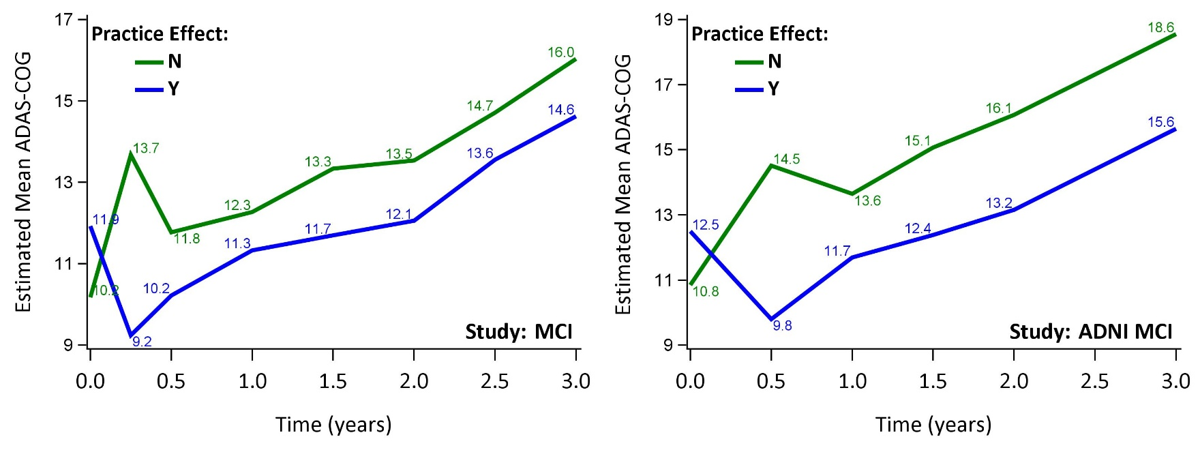

Supplement: S2 Fig — The practice effects group progressed more slowly than the non-practice effects group. (DOCX) [file pone.0228064.s002.docx]

S3 Fig


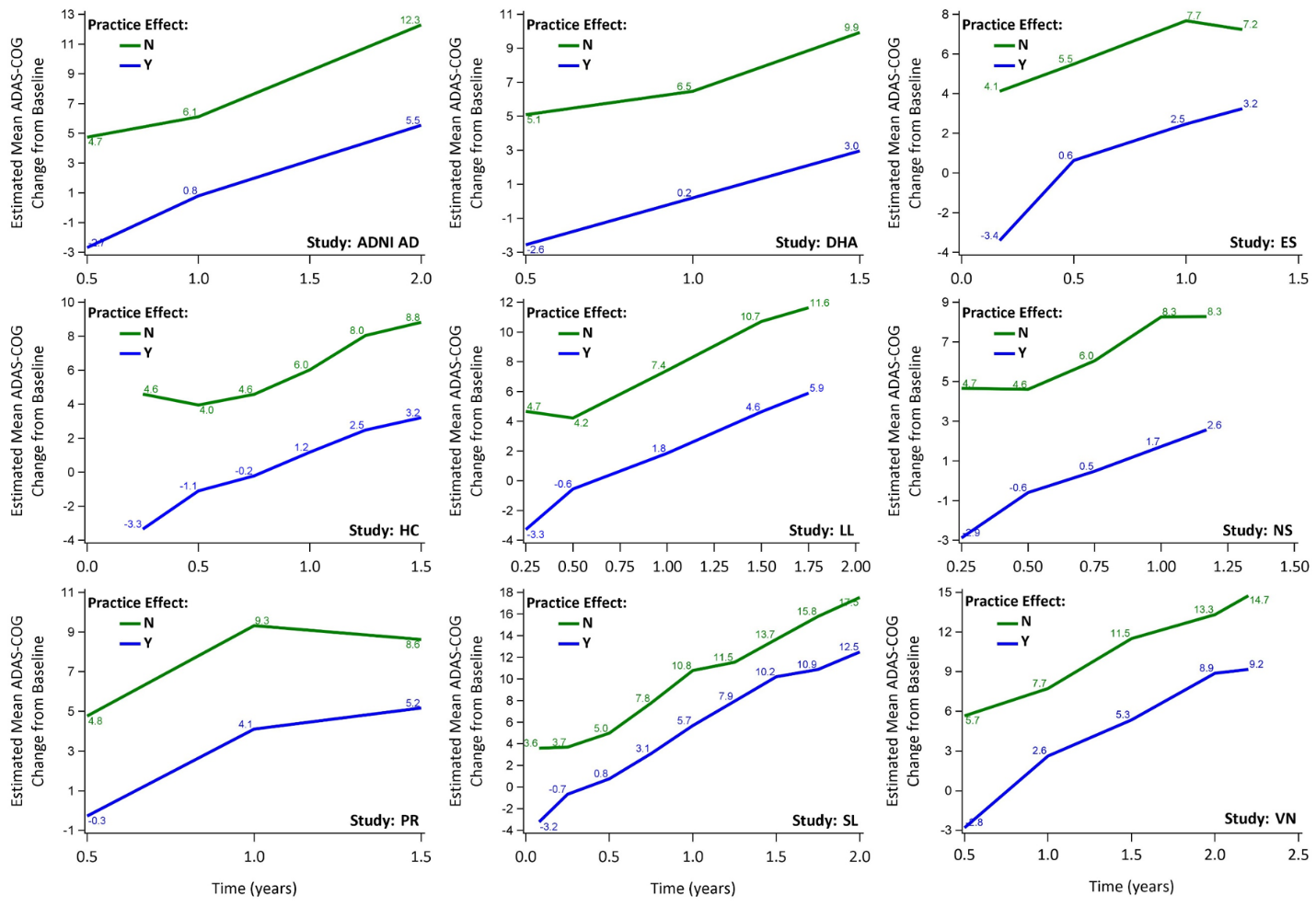

Supplement: S3 Fig — The practice effects group progressed more slowly than the non-practice effects group in all studies. (DOCX) [file pone.0228064.s003.docx]

S4 Fig


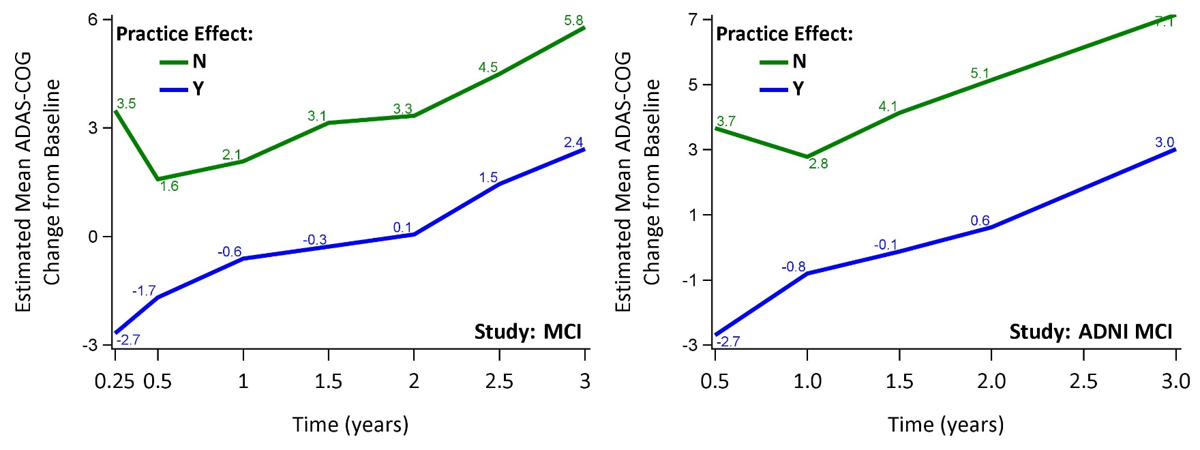

Supplement: S4 Fig — The practice effects group progressed more slowly than the non-practice effects group. (DOCX) [file pone.0228064.s004.docx]

S5 Fig


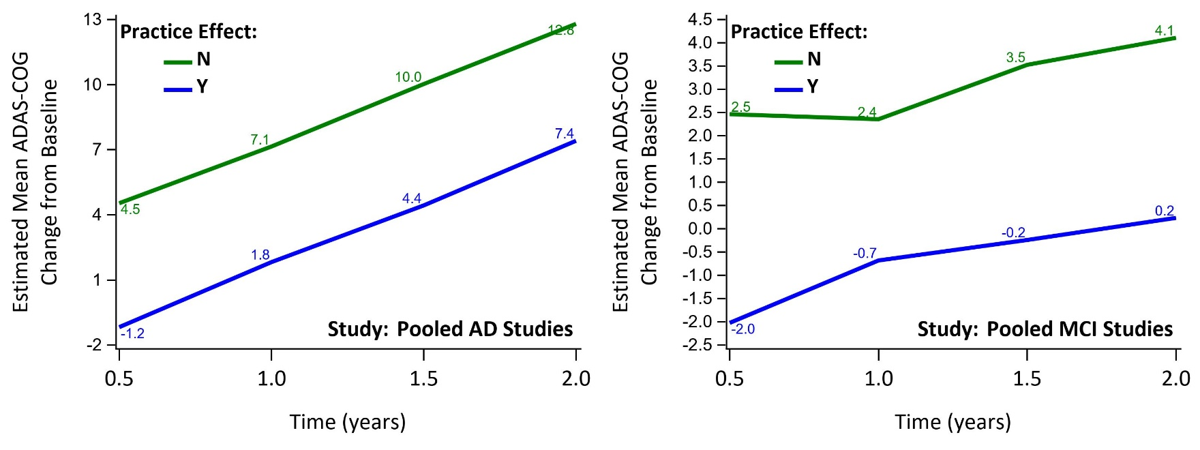

Supplement: S5 Fig — Similar results to those individual studies were observed for the mean ADAS-Cog11 change over time. (DOCX) [file pone.0228064.s005.docx]

S6 Fig

**
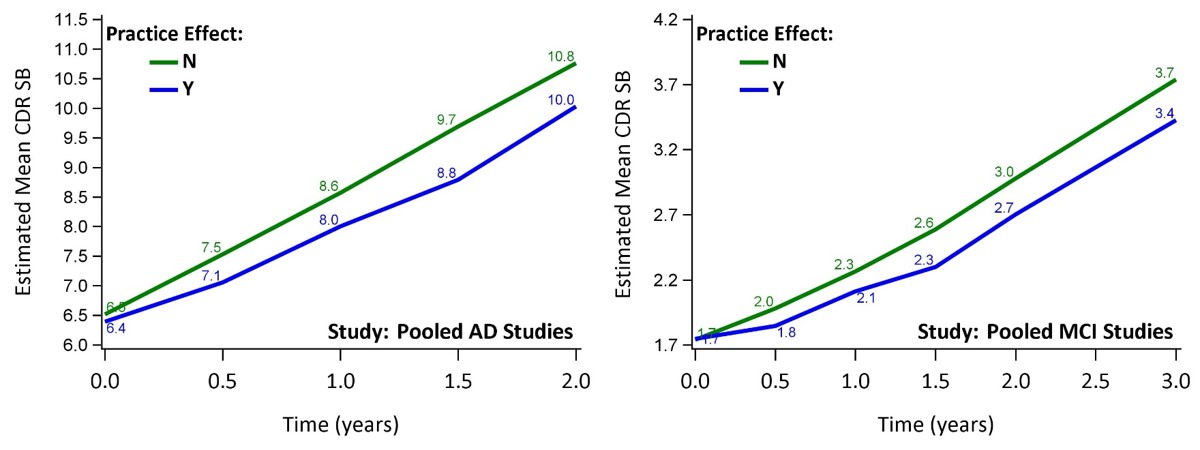
**

Supplement: S6 Fig — For both AD and MCI studies, the non-practice effects group declined faster. (DOCX) [file pone.0228064.s006.docx]

S7 Fig


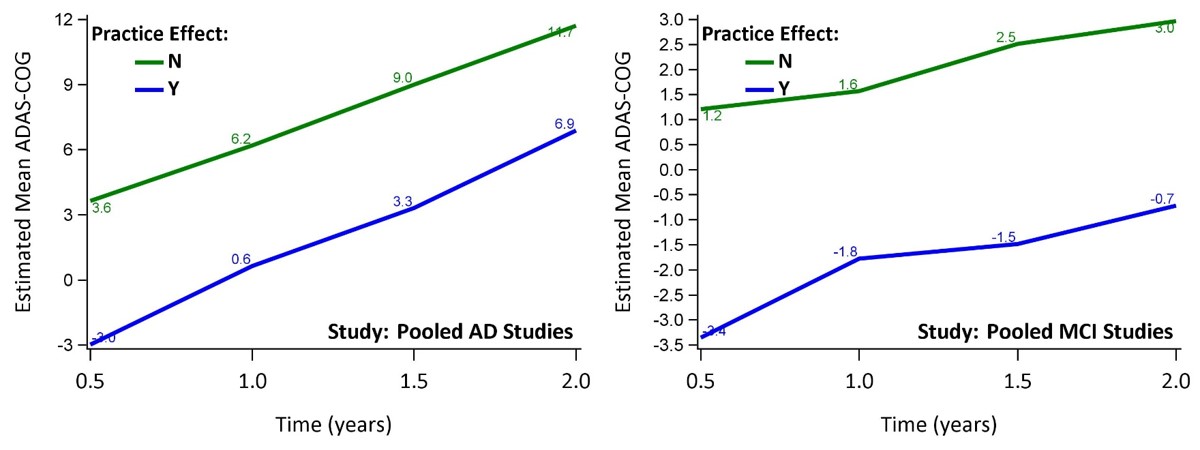

Supplement: S7 Fig — (DOCX) [file pone.0228064.s007.docx]
